# Does development of submucosal dissection models influence quality of training? Comparison of existing models

**DOI:** 10.1055/a-2621-5244

**Published:** 2025-06-17

**Authors:** Clara Yzet, Jérémie Jacques, Pierre Lafeuille, Jérémie Albouys, Jean-Baptiste Chevaux, Emmanuel Coron, Stanislas Chaussade, Sarah Leblanc, Vincent Lepilliez, Thimothee Wallenhorst, Thierry Ponchon, Jérôme Rivory, Romain Legros, Michel Lefranc, Marion Schaefer, Mathieu Pioche

**Affiliations:** 1 36609 Gastroenterology, Hopital Edouard Herriot, Lyon, France; 2 36673 Gastroenterology, Centre Hospitalier Universitaire Amiens-Picardie, Amiens, France; 3 Service d'Hépato-gastro-entérologie, CHU Dupuytren Limoges, Limoges, France; 4 36609 Gastroenterology, Groupement Hospitalier Edouard Herriot, Lyon, France; 5 37925 Hepato-gastro-entérologie, Hopital Dupuytren, Limoges, France; 6 26920 Department of Gastroenterology, Nancy Regional University Hospital Center, Nancy, France; 7 27045 Gastroenterology Unit, Nantes University, Nantes, France; 8 Gastroenterology, Cochin Hospital, Paris, France; 9 89686 Gastroenterology, Jean Mermoz Private Hospital, Lyon, France; 10 89686 Gastroenterology, Hôpital Privé Jean Mermoz, Lyon, France; 11 36684 Department of Endoscopy and Gastroenterology, University Hospital Centre Rennes, Rennes, France; 12 Hepatogastroenterology, Edouard Herriot, LYON, France; 13 Gastroenterology, Edouard Herriot Hospital, Lyon, France; 14 Service d'Hépato-gastro-entérologie, CHU Dupuytren, Limoges, France; 15 36673 Neurologic Surgery, Centre Hospitalier Universitaire Amiens-Picardie, Amiens, France; 16 26920 Hepato-gastroenterology, Centre Hospitalier Universitaire de Nancy, Vandoeuvre les Nancy, France

**Keywords:** Endoscopy Lower GI Tract, Endoscopic resection (polypectomy, ESD, EMRc, ...), Quality and logistical aspects, Training

## Abstract

**Background and study aims:**

Use of endoscopic submucosal dissection (ESD) is growing, but access to it remains limited. The aim of this study was to compare the performance of various existing models and the progress made by students on them.

**Methods:**

Four training models (bovine colon (ex-vivo), ex vivo porcine model, live porcine model, and an artificial model (Endogel)) were evaluated during a 1-week training course. Each participant was evaluated at the beginning (D1) and at the end of the training (D5). Learners performed a standardized ESD of 2 cm on the four models in a randomized order. Experts evaluated the ability of participants to perform ESD using the objective structured assessment of technical skill score (OSATS).

**Results:**

Sixteen students were involved, the average age was 35.6 years (+/- 4.6) and they practiced endoscopy for 10 years (+/-5.3). The OSATS significantly increased in each model during the week, with mean scores increasing from 8.6 to 23.3, from 10.7 to 12.9, from 8.8 to 21.3 and from 8.2 to 12.5 for the bovine colon, ex vivo porcine model, live porcine model, and Endogel models, respectively.

**Conclusions:**

Ex-vivo models are good models for learning ESD skills. The bovine colon model seems to be the most discriminating. Synthetic models should be reserved for novices.

## Introduction

The indication for endoscopic submucosal dissection (ESD) is expanding across all segments of the digestive tract but its adoption in Western countries is still progressing at a slow pace. ESD necessitates a heightened level of endoscopic proficiency and mandates rigorous training, even for experienced endoscopists. The learning curve for colonic ESD is long. R0 resection rates during the initial learning phase range from 46% to 75%, accompanied by an elevated incidence of complications (20% perforations and 20% conversions to surgery) 1 2 3 4 .

Several factors have been identified to explain the rate of complications, including operator expertise and volume of the ESD center 5 6 . Pre-practice utilization of animal models has been shown to decrease the complication rate and increase the en bloc resection rate. The European Society of Gastrointestinal Endoscopy (ESGE) recently issued a statement on ESD practice 7 . They recommend a minimum of 20 ESD procedures on models before transitioning to human subjects, with the objective of achieving at least eight en bloc complete resections in the last 10 training cases, and with no perforation 8 .

A primary obstacle to wider adoption of ESD in Western countries is the challenge in accessing animal models. Live animal models offer an advantage due to their similarity to humans, allowing for replication of typical operating conditions encountered in real practice, including the potential to mimic respiratory movements and address common complications in real time. Ex vivo models, such as bovine colon and ex vivo porcine model, are also available but are considered less realistic. Use of animal models is constrained by ethical and societal considerations, the theoretical risk of zoonosis, their accessibility, cost, and ethical concerns about sacrificing animals after each session.

To address these challenges, ESD simulators have been developed to facilitate learning various stages of ESD. They have the potential to enhance skill acquisition and technique implementation, thereby reducing training costs and the number of animals sacrificed. The objective of this study was to compare different ESD models available in France during a national ESD training week in terms of user opinion and progression capability.

## Methods

### Description of ESD training week

Since 2021, the French society of endoscopy has organized an ESD training program. During the year, a theoretical course in ESD is offered to 16 trainees previously selected because of their motivation and the local possibility of ESD companionship. After the theory classes, a practical training course of 5 days is organized to learn from the expert during simulation on ESD models.

### Organization of the evaluation

We designed a prospective evaluation of the practice of simulated ESD on four different ESD models during this week to compare the training potential and the user opinion on Day 1 and Day 5 of the training week for each model.

Four training models (bovine colon [ex vivo], ex vivo porcine model, live porcine model and an artificial model [Endogel]) were evaluated during this 1-week training course.

**Fig. 1:**
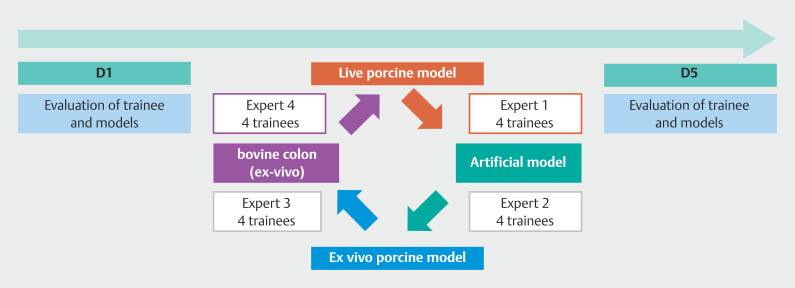
ESD training organization and evaluation.

Each participant was evaluated at the beginning (D1) and at the end of their training (D5). Four groups of four participants were created. In each group, the same ESD knife (Splash M Knife, Flush Knife, Gold Knife, Dual J Knife) was used to train on the four ESD models. Each group rotated among the four models and was evaluated at D1 and D5 ( Fig. 1 ). The running order was randomized at D1 and D5.

Between the two evaluations, the trainees had intensive ESD formation supervised by French ESD experts on animal models. This training was conducted on live porcine, bovine colon, and ex vivo porcine models and focused exclusively on ESD techniques, including circumferential incision, cutting, and dissection with or without traction. Under the supervision of ESD experts, students received guidance and correction as needed, covering aspects such as scope positioning and knife handling.

### Standard ESD procedure

In each model, the trainees had to perform a 20-mm ESD. A simulated lesion was done by the expert in each model using a snare applied on the mucosa with force coagulation current in order to provide a whitish oval simulated lesion. Then, the trainees had to perform submucosal injection of the lesion (using a saline solution colored with indigo carmine), incision of the lesion to gain access to the submucosae, and the dissection phase without traction. They all had10 minutes on each model to perform the entire resection.

### Evaluation of the trainees and models

Experts evaluated the ability of each participant using an objective structured assessment of technical skill (OSATS) 9 . We modified this score for endoscopic evaluation ( **Supplementary Table 1** ). Information about complete incision, submucosal access, and total dissection also was collected by the experts. For each model, each expert his impressions about conductivity, slide, softness, and visualization of the submucosae.

Level of satisfaction with each model was assessed using a numerical scale ranging from 0 (poor) to 10 (excellent).

At the end of the week, the expert graded a student's ability to perform ESD on a scale from 0 to 20 ( **Supplementary Table 2** ).

### Statistical analysis

Quantitative variables were calculated as mean, (interval) or median (interquartile range), and qualitative variables were calculated as a percentage.

To evaluate the progression of the student on each model during the ESD training week, we first hypothesized that the training during the week did not differ between students. The normality was verified with the Shapiro-Wilk test. We then compared the difference between the OSATS obtained by the students on D1 and D5 on each of the models using the Student's paired *t* -test. The percentage of complete incision, submucosae access, and full dissection were also tested between D1 and D5 using with the Fisher's exact test.

Ability to perform ESD was evaluated using a visual analogue scale ranging from 0 to 20. Pearson's coefficient was used to assess the correlation between the note obtained on the visual analogue scale and the OSATS. Correlation was judged very strong from 1 to 0.9, strong from 0.9 to 0.7, moderate from 0.7 to 0.5, low from 0.5 to 0.3. and poor from 0.3 to 0. The alpha risk was set to 0.05.

### Ethics

Use of animal models was approved by the local committee of Limoges simulation platform (EMIS, Limoges, France).

## Results

### Trainee characteristics

Sixteen students were involved, most of whom were male (11/16, 68%) with an average age of 35.6 years (+/- 4.6) and had been practicing endoscopy for an average of 10 years (+/-5.3). One-quarter of participants had never performed an ESD, 56.2% had performed fewer than 10, and one participant reported performing more than 20 ESDs.

### Skills abilities and progression

**Fig. 2:**
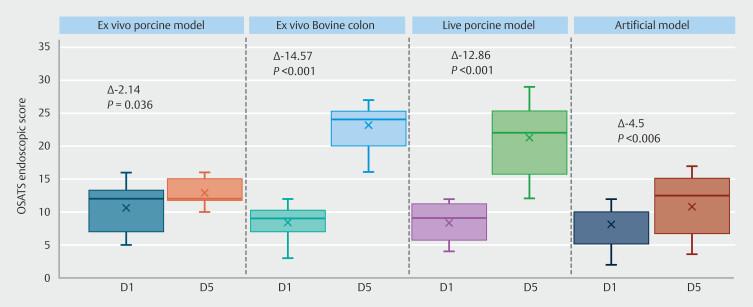
Progression of the OSATS endoscopic score during the ESD week on each model.

The OSATS was evaluated for each participant at D1 and D5 and statistically improved on each model between D1 and D5 ( Fig. 2 ).

Mean OSATS at D1 and D5 with the ex vivo porcine model were 10.71 (SD 3.5) and 12.86 (SD 2.03), respectively, with a mean difference of -2.14 (SD = 3.44; 95% confidence interval [CI] -4.06 to -0.23; *P* = 0.036).

For the bovine colon, mean OSATS at D1 and D5 were 8.57 (SD 2.59) and 23.14 (SD 3.11), respectively, with a mean difference of -14.57 (SD 3.72; 95% CI -16.64 to -12.5; *P* < 0.001).

Mean OSATS at D1 and D5 with a live porcine model were 8.43 (SD 2.98) and 21.29 (SD 5.33), respectively, with a mean difference of -12.86 (SD 5.53; 95% CI -15.93 to -9.78; *P* < 0.001).

For artificial model, the mean OSATS at D1 and D5 were 8.19 (SD 3.49) to 12.5 (3.14), respectively, (SD 5.0, Δ -4.5; SD 4.6; 95% CI -7.5 to -2.5; *P* = 0.006). Overall performance and procedure flow significantly improved in all models, except for the artificial model. Results of each subitem in the OSATS are detailed in Table 1 .

**Table:** **Table 1** Detailed mean scores for each item on the objective structured assessment of technical skill (OSATS) across models.

	Respect for tissue		P value	Time and motion		P value	Instrument handling		P value	Control knob		P value	Flow operation		P value	Overall performance		P value
	**D1**	**D5**		**D1**	**D5**		**D1**	**D5**		**D1**	**D5**		**D1**	**D5**		**D1**	**D5**	
Ex vivo porcine	**3.0**	**3.8**	**0.041**	**2.6**	**3.6**	**0.017**	**2.7**	**3.9**	**0.001**	3.7	3.5	0.56	**3.1**	**4.6**	**0.0036**	**2.5**	**3.3**	**0.017**
Bovine colon	**2.5**	**4.1**	**0.0001**	3.4	3.6	0.66	3.8	3.6	0.55	3.8	3.8	0.91	**2.5**	**4.3**	**< 0.001**	**1.7**	**3.8**	**< 0.001**
live porcine model	2.6	3.4	0.064	2.9	3.6	0.11	**2.6**	**3.6**	**0.0033**	3.9	3.5	0.26	**2.4**	**3.6**	**< 0.001**	**2.3**	**3.4**	**< 0.001**
artificial model	**2.5**	**3.3**	**0.0057**	**2.7**	**3.6**	**0.044**	3.3	3.9	0.089	3.4	3.8	0.35	2.6	3.2	0.16	2.4	2.8	0.21

The percentage of complete incision, submucosae access, and full dissection are presented in Table 2 .

**Table:** **Table 2** Skills abilities and progression on each model during the week.

	Complete incision			Submucosae access			Full dissection			Mean OSAT score		
	**D1**	**D5**	***P* value **	**D1**	**D5**	***P* value **	**D1**	**D5**	***P* value **	**D1**	**D5**	***P* value **
Ex vivo pig stomach	56.25%	56.25%	1	62.5%	31.25%	0.15	6.25%	0	1	**10.71**	**12.86**	**0.036**
Ex vivo cow colon	**18.75%**	**93.75%**	**0.019**	**12.5%**	**81.25%**	**< 0.001**	0%	0	1	**8.57**	**23.14**	**< 0.001**
live porcine model	68.75%	62.5%	1	37.5%	56.25%	0.48	6.25%	6.25%	1	**8.43**	**21.29**	**< 0.001**
artificial model	100%	87.5%	0.48	75%	75%	1	31.25%	18.75%	0	**8.19**	**12.50**	**0.006**
OSATS, objective structured assessment of technical skill.												

### Experts’ impressions

**Fig. 3:**
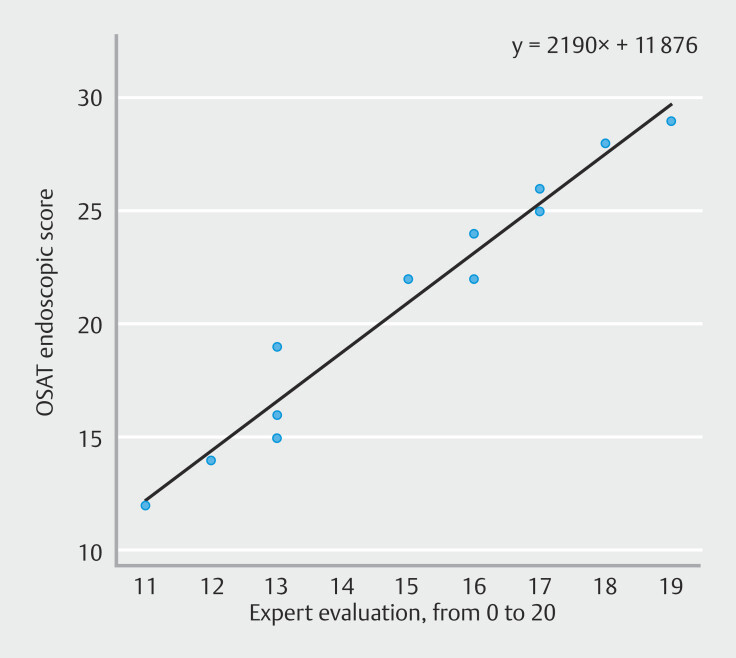
Correlation between expert impression of the ability of the student and the OSATS score.

The impression of conductivity, slide, softness, and visualization of submucosae were analyzed by the four experts ( **Supplementary Fig. 1** ). Experts rated each student at the end of the ESD week depending on their ability to practice ESD and the median score was 16 of 20 (IQR 14.5–17). This subjective appreciation correlates with the OSATS (ρ = 0.98; r ^2^ = 0.958; *P* < 0.001) ( Fig. 3 ).

### Satisfaction

**Fig. 4:**
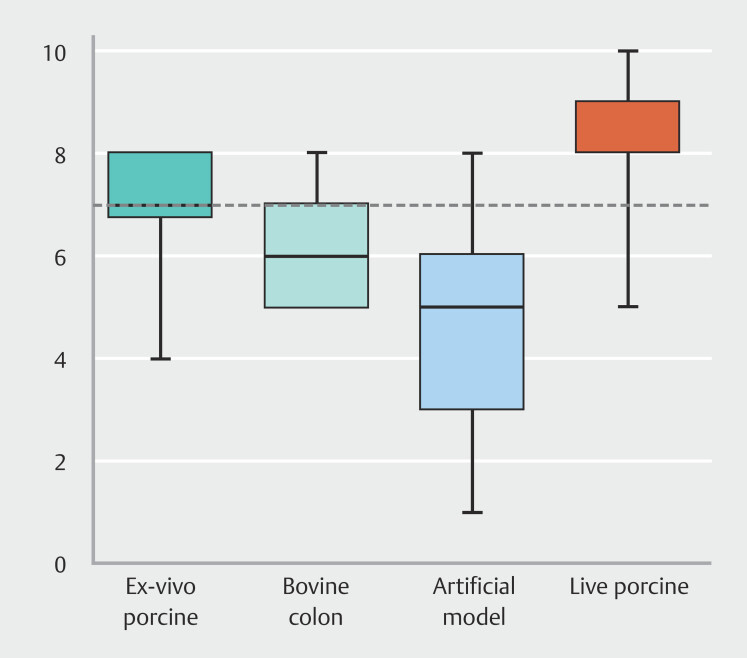
Trainee satisfaction level with the different endoscopic submucosal dissection training models.

Students overwhelmingly favored the live porcine model, with a median numerical scale rating of 8 (interquartile range [IQR] 7.5–8.7) ( Fig. 4 ). Students identified the live pig model as the sole model with which they were capable of effectively managing bleeding complications. All participants perceived practicing on living animal models as beneficial to their education.

## Discussion

Although ESD is the best option for obtaining R0 resection, beginning ESD on humans without adequate training is associated with low effectiveness and high mortality rates 10 . It requires multifaceted skill training, including simulation in various models to complete the long learning curve.

Our study demonstrated that the four simulation models tested are effective for obtaining a progression. All trainees acquired skills and, therefore, progressed during the training week. But the four models are not equivalent. In our study, a synthetic model allowed easy incision and submucosal access, even for beginners. The rate of achieving full dissection with this model exceeded that of other existing models, yet the progression in the OSATS on it was relatively modest. On each model, precision of the gesture assessed by the “respect for tissue” item improved at the end of the training week. The resection technique was statistically more fluid at the end of the week for all three animal models. The artificial model on each item showed a less significant difference between the beginning and the end of the training week. Consequently, based on our finding, the synthetic model does not seem sufficient to discriminate trainees depending on their progression. However, it emerged as the optimal choice for initial skill acquisition and introduction to ESD techniques. The advent of synthetic models raises questions about the actual need for animal sacrifice in training sessions and the potential for expanding the number of ESD practitioners 11 . However, limitations were noted, such as managing complications like bleeding and perforation, ensuring optimal visibility of the submucosal layer using gravity, and controlling air with insufflation and suction during the procedure. Recently, Mitsui et al. reported a high satisfaction and acceptability rate among experts using a new training model 12 .

Animal models, particularly the bovine colon, presented higher difficulty levels in ESD. The bovine colon model appears to be the most effective in discriminating the progression of students, because it exhibited the highest delta observed on the OSATS. This may be attributed to the challenging accessibility of the submucosae in this model due to avascular tissue and the freezing process, which can compromise tissue quality. Although the bovine colon is not the preferred model according to our subjective evaluation by the students, it could potentially be the optimal choice due to factors such as accessibility, cost-effectiveness, and absence of additional animal sacrifices. This model holds promise for evaluating ESD progression in studies that compare different ESD training strategies.

A live porcine model had the best satisfaction for students and a good discrimination potential but it poses challenges for widespread implementation in training centers, especially due to factors such as animal sacrifice, cost, and facility requirements. Although it brings theoretical advantages such as bleeding, respiratory movement, perforation management, the potential for discrimination does not surpass the bovine colon and ethical concerns, animal welfare, and the theoretical risk of zoonosis need to be addressed with this model.

In our study, we tried to evaluate skill progression using the OSATS endoscopic score, derived from the surgical training skills evaluation. The OSATS is complex to evaluate but highly correlated to the expert impression of trainee capacity.

If the models are important to learn first ESD skills, supervision is mandatory for the first human procedure. If supervision by an expert was the standard until now, virtual supervision has already demonstrated its own benefit but could be improved by artificial intelligence (AI) 13 14 . Recently, Cao et al. pioneered the creation of AI capable of recognizing different stages of ESD. Envisioning the future, there is optimism for development of an AI system that can guide practitioners on where to incise the lesion, thereby preventing complications and reducing the learning curve for ESD.

Our study has some limitations. First, we did not evaluate traction strategies in the four models while use of ESD is growing. Although all the models tested can be employed with traction 15 , this study aimed to evaluated initial ESD performance without adding the complexity of traction strategy and its own learning curve. Second, the participants were evaluated on D1 and D5 by the same evaluators who trained them during the entire week, which could have biased the results. The intensive week of training was conducted using live porcine and ex vivo models and synthetic models were not utilized due to their cost. Although the results obtained on D1 and D5 may be subject to bias from this limitation, each student received an equal amount of training time on the animal models, which are inherently more challenging than synthetic models. The standardized structure of the intensive program likely helped mitigate this potential bias. The experts were also not blind during the final evaluation, and use of a modified OSATS for endoscopic evaluation can be criticized because it is not yet validated. Finally, this study demonstrated the high potential of an animal model to obtain student progression, but correlation between animal skill enhancement and first human procedures is still missing. That evaluation is ongoing in a trainee long-term follow-up study.

## Conclusions

In conclusion, although synthetic models offer a valuable opportunity for early beginners to initiate ESD training, animal models remain essential for accurately measuring trainee progression. The cow colon model stands out for its discriminative capacity, accessibility, and absence of additional animal sacrifices. Although the living pig model remains the preferred option for trainees due to its realistic features, ethical considerations and cost concerns associated with it could raise questions in training workshops. Therefore, a balanced approach that integrates both synthetic and animal models may offer the most comprehensive and effective ESD training experience while addressing ethical and practical considerations.

## Supplementary Material

Supplementary Material Zusatzmaterial
