# Supplementary material for: Does development of submucosal dissection models influence quality of training? Comparison of existing models
Source: Endosc Int Open. 2025 Jun 17;13:a26215244. doi: 10.1055/a-2621-5244 (PMC12223905; doi:10.1055/a-2621-5244)

Thieme

Supplementary Table 1 Objective structured assessment of technical skill (OSATS) endoscopic score.

Score	Respect for tissue	Time and motion	Instrument handling	Control knob	Flow operation	Knowledge of procedure	Overall performance
1	Caused tissue damage	Unnecessary moves	Inappropriate instrument use	Inappropriate use of the control knob	Seemed unsure of the next move	Insufficient knowledge	Very poor
2	-	-	-	-	-	-	-
3	Occasionally caused damage	Some unnecessary moves	Occasionally stiff or awkward	Some unnecessary use	Some forward planning	Knew all important steps	Competent
4	-	-	-	-	-	-	-
5	Minimal tissue damage	Economy of movement	Fluid movements	Appropriate use	Planned operation	Familiarity with all steps	Clearly superior

Thieme

Supplementary Table 2 Rating by the expert from 0 to 20 on student ability to perform ESD.

Rating	Meaning
0 to 5	The student is not ready to begin performing ESD on humans
> 5 to 10	The trainee still needs training and strong supervision before beginning ESD on humans
> 10 to 15	The student is able to begin performing ESD on humans under supervision
> 15	The student is able to begin performing ESD autonomously on humans

Supplementary Fig. 1 Experts' impressions of the different models.

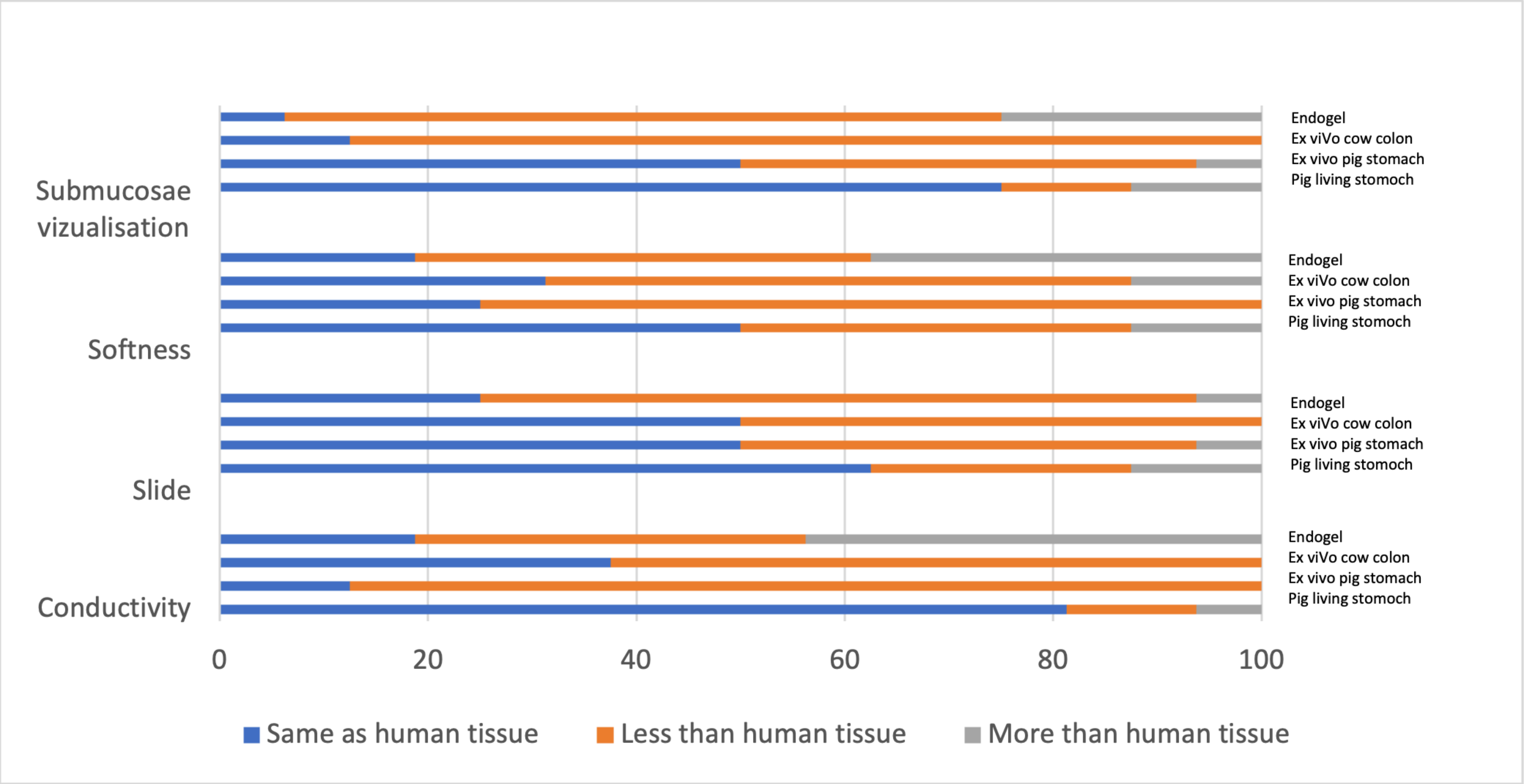

Supplement: Supplementary file 1 — Supplementary Material [file 10-1055-a-2621-5244_26241570.pdf]
